# Illuminating the Sites of Enterovirus Replication in Living Cells by Using a Split-GFP-Tagged Viral Protein

**DOI:** 10.1128/mSphere.00104-16

**Published:** 2016-07-06

**Authors:** H. M. van der Schaar, C. E. Melia, J. A. C. van Bruggen, J. R. P. M. Strating, M. E. D. van Geenen, A. J. Koster, M. Bárcena, F. J. M. van Kuppeveld

**Affiliations:** a Virology Division, Department of Infectious Diseases and Immunology, Faculty of Veterinary Medicine, Utrecht University, Utrecht, The Netherlands; b Department of Molecular Cell Biology, Section Electron Microscopy, Leiden University Medical Center, Leiden, The Netherlands; University of Pittsburgh School of Medicine

**Keywords:** correlative light electron microscopy, enterovirus, live-cell imaging, viral replication

## Abstract

Like all other positive-strand RNA viruses, enteroviruses generate new organelles (replication organelles [ROs]) with a unique protein and lipid composition on which they multiply their viral genome. Suitable tools for live-cell imaging of enterovirus ROs are currently unavailable, as recombinant enteroviruses that carry genes that encode RO-anchored viral proteins tagged with fluorescent reporters have not been reported thus far. To overcome this limitation, we used a split green fluorescent protein (split-GFP) system, comprising a large fragment [strands 1 to 10; GFP(S1-10)] and a small fragment [strand 11; GFP(S11)] of only 16 residues. The GFP(S11) (GFP with S11 fragment) fragment was inserted into the 3A protein of the enterovirus coxsackievirus B3 (CVB3), while the large fragment was supplied by transient or stable expression in cells. The introduction of GFP(S11) did not affect the known functions of 3A when expressed in isolation. Using correlative light electron microscopy (CLEM), we showed that GFP fluorescence was detected at ROs, whose morphologies are essentially identical to those previously observed for wild-type CVB3, indicating that GFP(S11)-tagged 3A proteins assemble with GFP(S1-10) to form GFP for illumination of bona fide ROs. It is well established that enterovirus infection leads to Golgi disintegration. Through live-cell imaging of infected cells expressing an mCherry-tagged Golgi marker, we monitored RO development and revealed the dynamics of Golgi disassembly in real time. Having demonstrated the suitability of this virus for imaging ROs, we constructed a CVB3 encoding GFP(S1-10) and GFP(S11)-tagged 3A to bypass the need to express GFP(S1-10) prior to infection. These tools will have multiple applications in future studies on the origin, location, and function of enterovirus ROs.

**IMPORTANCE** Enteroviruses induce the formation of membranous structures (replication organelles [ROs]) with a unique protein and lipid composition specialized for genome replication. Electron microscopy has revealed the morphology of enterovirus ROs, and immunofluorescence studies have been conducted to investigate their origin and formation. Yet, immunofluorescence analysis of fixed cells results in a rather static view of RO formation, and the results may be compromised by immunolabeling artifacts. While live-cell imaging of ROs would be preferred, enteroviruses encoding a membrane-anchored viral protein fused to a large fluorescent reporter have thus far not been described. Here, we tackled this constraint by introducing a small tag from a split-GFP system into an RO-resident enterovirus protein. This new tool bridges a methodological gap by circumventing the need for immunolabeling fixed cells and allows the study of the dynamics and formation of enterovirus ROs in living cells.

## Abstract (precis)

Enteroviruses induce the formation of membranous structures (replication organelles [ROs]) with a unique protein and lipid composition specialized for genome replication. Electron microscopy has revealed the morphology of enterovirus ROs, and immunofluorescence studies have been conducted to investigate their origin and formation. Yet, immunofluorescence analysis of fixed cells results in a rather static view of RO formation, and the results may be compromised by immunolabeling artifacts. While live-cell imaging of ROs would be preferred, enteroviruses encoding a membrane-anchored viral protein fused to a large fluorescent reporter have thus far not been described. Here, we tackled this constraint by introducing a small tag from a split-GFP system into an RO-resident enterovirus protein. This new tool bridges a methodological gap by circumventing the need for immunolabeling fixed cells and allows the study of the dynamics and formation of enterovirus ROs in living cells.

## INTRODUCTION

The *Enterovirus* genus of the *Picornaviridae* family comprises many human pathogens, such as poliovirus, coxsackievirus A and B, enterovirus 68, enterovirus 71, and rhinovirus, which can cause a wide spectrum of illnesses (1). Being obligate intracellular parasites, enteroviruses rely on the machineries of their host cell for propagation. Like all other viruses that carry a positive-sense, single-stranded RNA genome, enteroviruses redecorate the cell’s interior to form new membranous structures that serve as a platform for viral RNA replication (2–6). These structures may aid in concentrating as well as in conferring the proper topology of all required components for genome replication. Furthermore, it has been suggested that they can shield viral RNA products from degradation by cellular RNases or from detection by sensors of the innate immune system (7, 8).

The morphology of the enterovirus-induced membrane structures (often termed replication organelles [ROs]) has been a subject of intense investigation. Two-dimensional electron microscopy (EM) studies have shown both single-membrane and double-membrane structures, depending on the cell type, time point, and experimental procedure (9–16). Recently, two studies with poliovirus and coxsackievirus B3 (CVB3) were conducted to reveal the three-dimensional (3D) structure of the ROs in the course of infection using electron tomography (17, 18). Both studies showed that the first structures detected upon infection are single-membrane tubular structures. These tubules appear to be formed at the expense of Golgi membranes, since in most cell sections, the Golgi apparatus is no longer detected when the tubules are present. The tubules emerge during the exponential phase of viral RNA replication, suggesting that they are the preeminent structures supporting viral genome synthesis. Later in infection, the tubular ROs morph into double-membrane vesicles (DMVs) and multilamellar structures, a phenomenon that is reminiscent of autophagy. It was shown for poliovirus that newly synthesized viral RNA localizes not only to the tubular structures but also to the DMVs, implying that the DMVs may also facilitate genome replication (17). In addition, DMVs have been proposed to mediate nonlytic release of progeny virions (19–21).

While EM analyses have provided insight into the structure of the enterovirus ROs, fluorescence microscopy studies have focused on unraveling their origin by investigating the presence of essential host factors or marker proteins on the ROs. These studies have been performed in cells that had been fixed at various time points postinfection, usually at 1- to 2-h intervals, which gives limited insight into the dynamics of RO formation. ROs are mostly visualized by immunolabeling using antibodies directed against viral proteins that are anchored in the RO membrane, i.e., 2B, 2C, or 3A. With this approach, ROs were shown to colocalize with several proteins involved in endoplasmic reticulum (ER)-to-Golgi transport (22–25) as well as with LC3, a protein involved in the autophagy pathway (11, 13, 19). However, ROs are not mere remnants of the early secretory pathway or constituents of the autophagy pathway. Instead, enteroviruses seem highly selective in hijacking components from these pathways to create completely new organelles with a unique protein and lipid composition optimized for genome replication (reviewed in reference 5).

Although we are learning more and more about the morphology and origin of enterovirus ROs by three-dimensional EM studies and by fluorescence microscopy of fixed cells, it has not been possible thus far to directly visualize ROs in living cells. Live-cell imaging can provide unprecedented insights into the dynamics of biological processes, including RO formation and viral effects on other cellular structures or organelles, and may capture rare or rapid events that may be missed in the analysis of fixed cells. Moreover, observations by immunolabeling of fixed cells may be compromised by artifacts induced during sample preparation and should be complemented by live-cell imaging (26). To date, enterovirus ROs have been monitored only indirectly in living cells that expressed a fluorescently labeled cellular protein, the Golgi-resident Arf1-RFP (red fluorescent protein [RFP]-labeled Arf1), as an RO marker (22). Yet, the use of a cellular protein as a RO marker may complicate investigations examining the transition from the Golgi apparatus to early enterovirus ROs. A better strategy for direct visualization of ROs in living cells is to label a viral protein involved in their formation. The enterovirus genome can accept coding sequences of foreign proteins (green fluorescent protein [GFP] [27, 28], proLC3 [16], Timer [29], and luciferase [27, 30] among others) at the start of the open reading frame before the capsid coding region. In addition, a poliovirus encoding a viral proteinase (i.e., the 2A protein) tagged with dsRed has been generated (31). However, 2A is not a bona fide RO marker, as it does not localize exclusively to ROs. Attempts to generate enteroviruses that encode fluorescently labeled, membrane-anchored viral proteins to illuminate ROs have thus far been futile, most likely because fusion of a fluorescent protein to an RO-anchored viral protein impaired its function or liberation from the polyprotein. The use of small genetically encoded tags may overcome this limitation, as evidenced by the successful generation of recombinant polioviruses that encode small epitope tags (such as hemagglutinin [HA], FLAG, and c-myc) in their 3A protein (32) and a recombinant CVB3 encoding an HA tag in its 2B protein (33).

In this study, we used a split-GFP system (34) to tag the 3A protein of CVB3, a small protein of 89 amino acids with a C-terminal hydrophobic domain that inserts into the RO membranes (35). This split-GFP system is based on superfolder GFP, a variant of GFP that folds better, matures more rapidly, and fluoresces more brightly than GFP (36). Like GFP, superfolder GFP has a beta barrel structure consisting of eleven β-strands, which in this system are split into a large fragment [strands 1 to 10; GFP(S1-10)] and a small fragment [strand 11; GFP(S11)] of only 16 amino acids. Separately, these two fragments are nonfluorescent. Green fluorescence is emitted only when they assemble into superfolder GFP (34). We show that tagging 3A with GFP(S11) does not affect its known functions when 3A is expressed alone. When introduced into the viral genome of CVB3, GFP(S11)-tagged 3A assembled with GFP(S1-10) to illuminate enterovirus ROs as shown by correlative light electron microscopy (CLEM). We illustrate the suitability of CVB3 encoding this split-GFP-tagged 3A for live-cell imaging by monitoring the development of ROs and the disintegration of the Golgi apparatus, as visualized using GM130-mCherry, in infected cells in real time. This new tool simplifies the visualization of enterovirus ROs, avoiding immunolabeling of viral proteins in fixed cells, and will have multiple applications in future studies on the origin, location, and function of enterovirus ROs.

## RESULTS

### 3A tagged with GFP(S11) assembles with GFP(S1-10) to yield GFP fluorescence.

**FIG 1:**
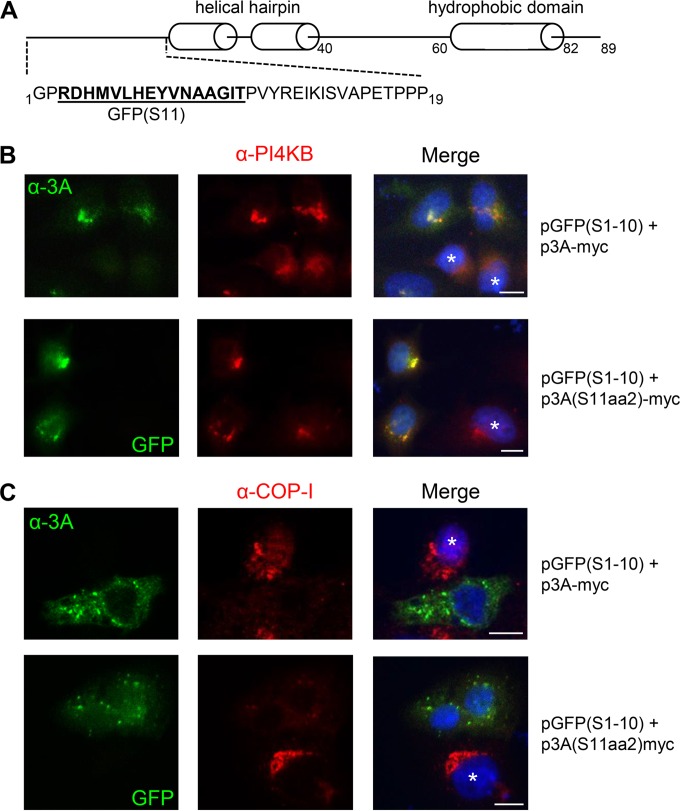
3A(S11aa2) recruits PI4KB and dissociates COP-I from Golgi membranes. (A) CVB3-3A(S11aa2) protein. The GFP(S11) tag (bold, underlined) is inserted after amino acid 2 of 3A without any linker residues. The hydrophobic domain, predicted by the Kyte and Doolittle method (see reference 66), is indicated as well as the location of the helical hairpin predicted by nuclear magnetic resonance (NMR) analysis of the truncated poliovirus 3A protein. (B and C) HeLa R19 (B) or BGM (C) cells were cotransfected with the plasmid encoding GFP(S1-10) [i.e., pGFP(S1-10)] and either p3A-myc or p3A(S11aa2)-myc. The next day, cells were fixed and subjected to immunofluorescence analysis. The 3A protein was visualized with an antibody staining for p3A-myc (a primary anti-3A [α-3A] antibody and a secondary Alexa Fluor 488-labeled antibody), while p3A(S11aa2) was visualized by the GFP fluorescence as a result of assembly with GFP(S1-10). PI4KB (A) and COP-I (B) were detected with immunofluorescence using a secondary Alexa Fluor 594-labeled antibody. Untransfected cells are indicated with a white asterisk. Nuclei were stained with DAPI. Wide-field images were acquired with an Olympus BX60 fluorescence microscope. Bars, 10 µm.

In order to visualize the enterovirus ROs in living cells, one of the nonstructural proteins that is anchored in the membranes of the ROs should be labeled with a fluorescent tag. A transposon-based insertion mutagenesis study revealed that the N-terminal region of poliovirus 3A tolerates small insertions (32, 37). Specifically, viable insertions were found after residues 2, 6, 9, 10, and 11. Since the 3A proteins of coxsackievirus B3 (CVB3) and poliovirus are highly homologous, these positions were good candidates for introduction of a small tag in CVB3 3A. We chose to insert GFP(S11) after amino acid 2 in CVB3 3A without using any linker residues, yielding 3A(S11aa2) (Fig. 1A).

Before introducing GFP(S11) into the viral genome, we first tested whether inserting the tag into 3A at this position would generate GFP fluorescence upon coexpression with GFP(S1-10). Staining with an anti-3A antibody demonstrated that the expression pattern of 3A(S11aa2) resembled that of untagged 3A [i.e., lacking GFP(S11)] in HeLa cells (see Fig. S1A in the supplemental material) and in BGM (buffalo green monkey) cells (data not shown), indicating that the GFP(S11) tag does not alter the localization of 3A. Coexpression of GFP(S1-10) with 3A(S11aa2), but not with untagged 3A, resulted in a GFP signal that greatly overlapped with the 3A pattern generated with the anti-3A antibody (see Fig. S1B, HeLa cells not shown). Furthermore, 3A(S11aa2) was not GFP fluorescent in the absence of GFP(S1-10) (see Fig. S1A). Thus, GFP(S1-10) can assemble with 3A(S11aa2) to yield GFP fluorescence at the sites where 3A is localized in the cell.

10.1128/mSphere.00104-16.1 Figure S1  Coexpression of 3A(S11aa2) with GFP(S1-10) yields GFP fluorescence. (A) Introduction of GFP(S11) into 3A does not alter its expression pattern. HeLa cells were transfected with plasmids encoding wild-type (wt) 3A (p3A-myc) or tagged 3A [p3A(S11aa2)-myc]. One day later, the 3A protein was stained with an antibody directed against 3A and a secondary Alexa Fluor 594-labeled antibody. (B) GFP fluorescence is detected only when GFP(S1-10) is coexpressed with 3A(S11aa2). BGM cells were cotransfected with the plasmid encoding GFP(S1-10) [pGFP(S1-10)] and either p3A-myc or p3A(S11aa2)-myc. One day later, the 3A protein was stained with a primary antibody directed against 3A and a secondary Alexa Fluor 594-labeled antibody. (A and B) Nuclei were stained with DAPI. Wide-field images were acquired with an Olympus BX60 fluorescence microscope. Bars, 10 µm. Download Figure S1, TIF file, 2 MB. Copyright © 2016 van der Schaar et al. 2016 van der Schaar et al. This content is distributed under the terms of the Creative Commons Attribution 4.0 International license.

### Introduction of GFP(S11) into 3A does not impair its known functions.

Next, we investigated whether 3A(S11aa2) is able to perform the same functions as untagged 3A. Enteroviruses hijack the Golgi-resident PI4KB (phosphatidylinositol 4-kinase IIIβ) via their 3A protein to ROs to enrich these membranes in PI4P (phosphatidylinositol 4-phosphate), an essential lipid for viral RNA replication (22). In line with previous observations (22, 38, 39), PI4KB was present in a faint Golgi-like pattern in untransfected HeLa cells, whereas in 3A-transfected cells, an intense PI4KB signal was detected at membranes containing untagged 3A (Fig. 1B). Likewise, in cells coexpressing 3A(S11aa2) and GFP(S1-10), PI4KB recruitment was observed to the membranes containing fluorescent 3A (Fig. 1B).

The enterovirus 3A protein interacts directly with GBF1, an essential host factor for enterovirus replication (27, 30). Under normal conditions, GBF1 is an activator of the small GTPase Arf1, which in turn recruits the COP-I complex to Golgi membranes upon activation (40). The COP-I coat initiates budding of transport vesicles that ferry cargo at the ER-Golgi interface and in the Golgi apparatus (41). Expression of 3A in isolation leads to the perturbation of ER-to-Golgi transport (30, 42–44), as demonstrated by the dissociation of COP-I from Golgi membranes in BGM cells (44), presumably as a result of the interaction of 3A with GBF1 (30, 44). Similar to untagged 3A, 3A(S11aa2) visualized by coexpression of GFP(S1-10) clearly caused COP-I dissociation (Fig. 1C).

In addition to the interaction with GBF1, enterovirus 3A also directly binds to the host factor ACBD3 (acyl coenzyme A [acyl-CoA]-binding protein domain 3), although the role of this protein in enterovirus replication is still enigmatic (38, 39, 45–47). We tested whether 3A(S11aa2) could still interact with GBF1 and ACBD3 in a mammalian two-hybrid system (38, 39). Similar to untagged 3A, 3A(S11aa2) interacted with GBF1 and ACBD3, even in the presence of overexpressed GFP(S1-10) (see Fig. S2 in the supplemental material).

Collectively, our results demonstrate that introduction of GFP(S11) in CVB3 3A after the second residue does not affect the ability of 3A to interact with previously identified host factors or to perform its known functions, which prompted us to introduce GFP(S11) into the viral genome of CVB3.

10.1128/mSphere.00104-16.2 Figure S2  3A(S11aa2) interacts with host factors GBF1 and ACBD3. (A and B) A mammalian two-hybrid assay was used to determine the ability of 3A(S11aa2) to interact with the N terminus of GBF1 (A) or ACBD3 (B). This assay was conducted in the absence of GFP(S1-10) (left graphs) or in the presence of GFP(S1-10) (right graphs) using full-length GFP as a transfection control. Bars represent the means of three samples ± standard deviations. Significant differences were calculated over the control sample with the highest value by a paired Student’s *t* test. ***, *P* < 0.001. Download Figure S2, TIF file, 1.1 MB. Copyright © 2016 van der Schaar et al. 2016 van der Schaar et al. This content is distributed under the terms of the Creative Commons Attribution 4.0 International license.

### Tagging 3A with GFP(S11) results in a replication-competent CVB3 that generates fluorescent replication organelles.

Introduction of the GFP(S11) tag into the infectious clone of CVB3 yielded viable virus, i.e., CVB3-3A(S11aa2). Sequence analysis of the viral genome confirmed that the tag was retained in the 3A protein without mutations. In addition, we generated a replication-competent CVB3 with the short affinity tag StrepII of 10 residues in 3A [i.e., CVB3-3A(StrepIIaa2)]. Insertion of the StrepII tag also did not affect the known functions of 3A (data not shown), and the tag was retained in 3A for five passages (data not shown), further demonstrating that residue 2 is an amenable site for introduction of a small tag.

**FIG 2:**
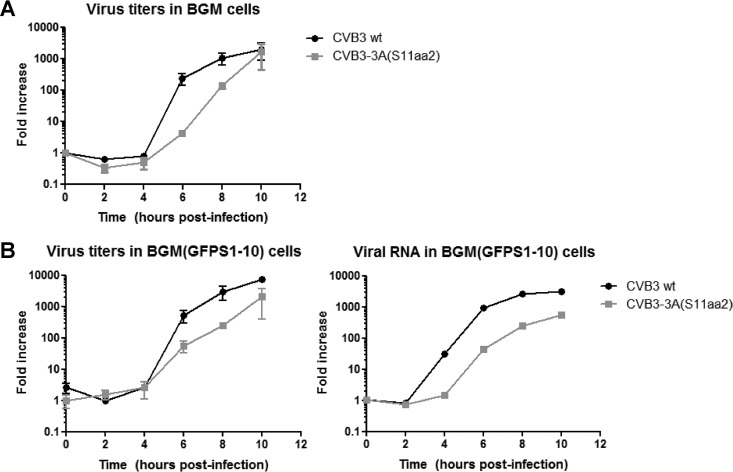
Single-cycle growth curve analysis of CVB3-3A(S11aa2). (A and B) BGM cells (A) or BGM cells that stably express GFP(S1-10) (B) were infected with wild-type (wt) CVB3 or CVB3-3A(S11aa2) for 30 min at an MOI of 1. At the time points indicated, cells were subjected to titration analysis after freeze-thawing cycles to determine the amount of infectious virus particles. Alternatively, cells were lysed to determine the amount of viral RNA by quantitative PCR. The results are expressed as fold induction relative to the quantities determined directly after removing the inoculum.

Subsequently, we tested whether the GFP(S11) tag in 3A affects the replication kinetics of the virus. Since cells that have been transfected with plasmid DNA are less susceptible to picornavirus infection (48), we generated single-cell clones of HeLa and BGM cells that stably express GFP(S1-10). Next, we compared the growth kinetics of CVB3-3A(S11aa2) to wild-type (wt) CVB3 in the presence and absence of GFP(S1-10). Samples were subjected to endpoint titration to determine the production of infectious virus or quantitative PCR to measure viral RNA levels. Replication of wt CVB3 in BGM cells and BGM(GFPS1-10) cells was nearly identical, showing that the presence of GFP(S1-10) did not alter CVB3 replication kinetics (Fig. 2A and B). The replication kinetics of tagged CVB3 in BGM(GFPS1-10) cells also resembled that of BGM cells, indicating that binding of GFP(S1-10) to 3A(S11aa2) does not impede replication (Fig. 2A and B). Both infectious virus production and viral RNA levels of the tagged CVB3 were delayed compared to those of wt CVB3 (Fig. 2B). This delay in replication has also been observed for recombinant polioviruses that encode 3A proteins with small epitope tags (32). Together, these findings showed that replication of CVB3-3A(S11aa2) is similar to other enteroviruses that encode small tags in a viral protein.

**FIG 3:**
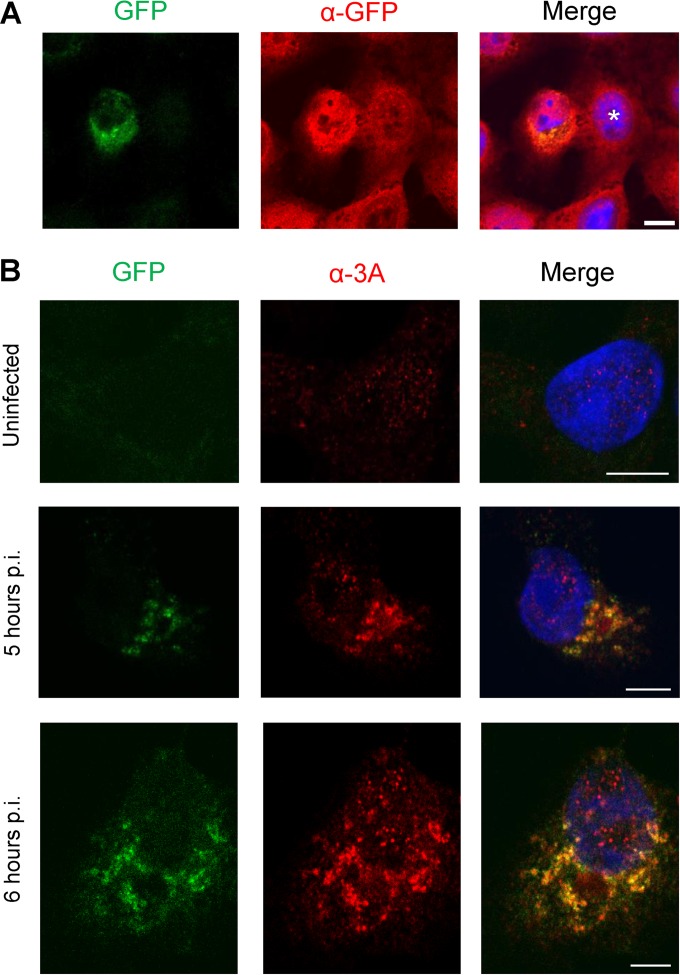
GFP fluorescence is emitted only from sites where 3A(S11aa2) is localized in cells. (A and B) BGM cells stably expressing GFP(S1-10) were infected with CVB3-3A(S11aa2). At 5 and 6 h p.i., cells were fixed and subjected to immunofluorescence analysis. The detected GFP fluorescence is the result of GFP(S1-10) assembling with 3A(S11aa2). Nuclei were visualized with DAPI. Images were acquired with a Leica SPE-II DMI-4000 confocal laser scanning microscope. (A) GFP(S1-10) was detected with a primary polyclonal antibody directed against GFP and a secondary Alexa Fluor 594-labeled antibody. The uninfected cell is marked with a white asterisk. (B) The 3A(S11aa2) protein was stained with a primary antibody directed against 3A and a secondary Alexa Fluor 594-labeled antibody. An uninfected cell and typical examples of cells early in infection (5 h p.i.) and later in infection (6 h p.i.) are shown. Bars, 10 µm.

Having produced replication-competent CVB3-3A(S11aa2), we then tested whether this virus induced GFP fluorescence in cells expressing GFP(S1-10). Figure 3 shows that these cells do emit GFP fluorescence upon infection with CVB3-3A(S11aa2). To investigate whether the GFP fluorescence was restricted to the sites where the 3A(S11aa2) proteins are localized in the cell, we first stained cells with an anti-GFP antibody. Figure 3A shows that the anti-GFP antibody recognized GFP(S1-10) as demonstrated by the cytoplasmic staining in uninfected cells, whereas BGM cells lacking GFP(S1-10) were negative (data not shown). In infected cells, GFP fluorescence was not observed throughout the cytoplasm but in a pattern that is reminiscent of a 3A staining pattern. This suggests that assembled, fluorescent GFP is found only at sites that contain 3A(S11aa2). Interestingly, while anti-GFP antibody staining in infected cells was detected throughout the cytoplasm, the signal was more intense at regions corresponding to GFP fluorescence. This is likely the result of recruitment of GFP(S1-10) to sites where 3A is localized. Another possibility is that the anti-GFP antibody has a higher affinity for full-length, assembled GFP.

To ensure that GFP fluorescence is emitted exclusively from 3A(S11aa2)-containing sites, cells from the same infection were stained with an anti-3A antibody. Figure 3B shows that GFP fluorescence was detected in a pattern that greatly overlapped with 3A visualized by immunofluorescence. However, the immunofluorescence with the 3A antibody generated a slightly more extensive 3A pattern, most likely as a consequence of not all 3A(S11aa2) proteins being bound by GFP(S1-10). This could be due to partial inaccessibility of 3A(S11aa2) proteins to their GFP(S1-10) counterparts, as 3A proteins localize to densely packed ROs.

**FIG 4:**
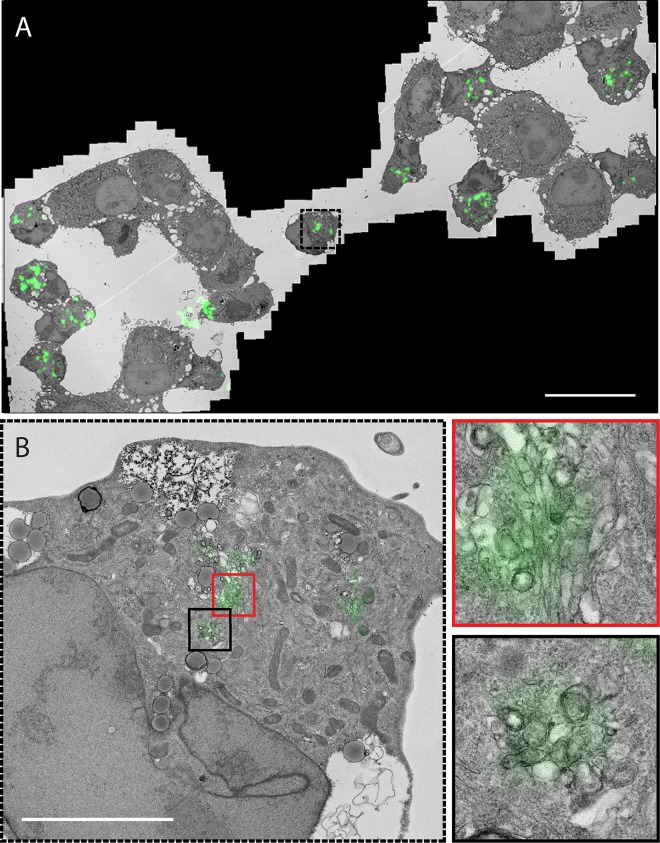
3A(S11aa2) localizes to ROs that have tubule and DMV morphologies. (A and B) BGM GFP(S1-10) cells were infected with CVB3-3A(S11aa2), stained with MitoTracker deep red FM, and monitored by live-cell imaging. Full-depth z-stacks of cells emitting GFP fluorescence were taken, and cells were processed for EM. Light microscopy (LM)-EM overlays were made using MitoTracker deep red FM as an orientation guide, and 3A-GFP signal was aligned to the corresponding EM image in this manner first at low magnification (A) and then within individual cells of interest (B) (panel B is the boxed area in panel A). 3A-GFP signal was found at the typical structures that develop during CVB3 infection, including single-membrane tubules (red box in panel B, enlargement shown on the right) and double-membrane vesicles (black box in panel B, enlargement shown on the right). Bars, 30 µm (A) and 5 µm (B).

Next, we set out to confirm that the emerging GFP fluorescence is emitted from ROs that contain 3A(S11aa2). To this end, we applied correlative light electron microscopy (CLEM) (49, 50), which provides a direct link between fluorescent signals and the virus-induced structures that underlie them at high resolution. The emerging fluorescent signal in BGM(GFPS1-10) cells infected with CVB3-3A(S11aa2) and stained with MitoTracker deep red FM was monitored by live-cell imaging. Cells were imaged and fixed at an intermediate time point in infection. Following sample processing and EM imaging, the MitoTracker signal and corresponding mitochondria were used to orient fluorescence and EM images with an independent marker. Using this method, 3A(S11aa2) was found to localize to ROs, evident as both tubules and clusters of DMVs and multilamellar structures (Fig. 4). Furthermore, the CLEM results not only show that GFP fluorescence was emitted from bona fide ROs, they also demonstrate that the GFP(S11) insertion in 3A does not affect the development of the different RO morphologies typically observed during enterovirus infections.

### Live-cell imaging reveals the dynamics of Golgi disassembly in live infected cells.

**FIG 5:**
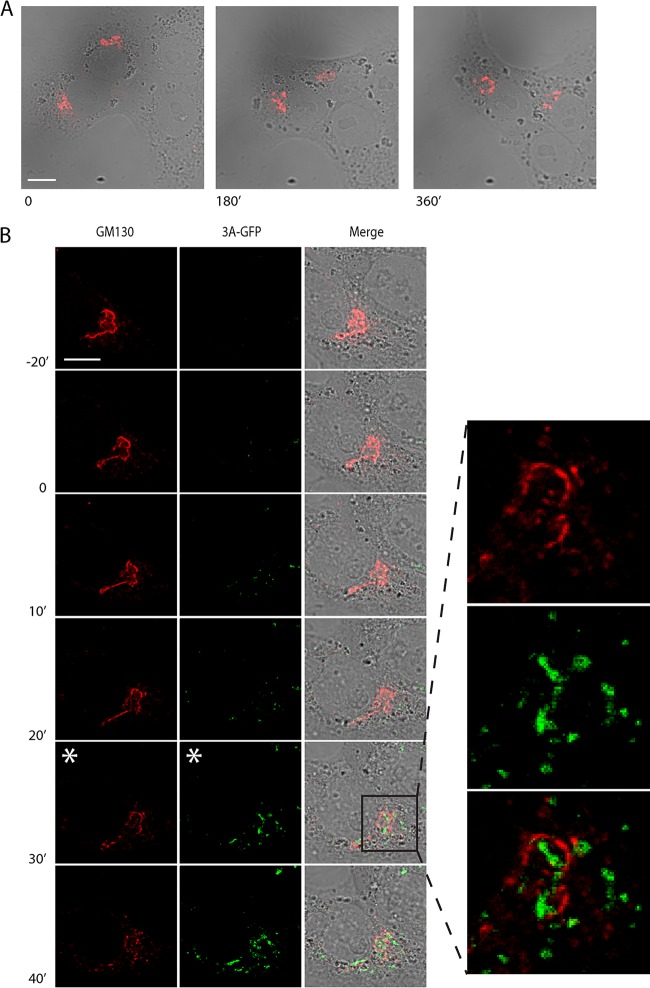
Live-cell imaging reveals the association between 3A accumulation and local perturbation to Golgi morphology. (A and B) BGM GFP(S1-10) cells transduced with mCherry-GM130 MLV particles were mock infected (A) or infected with CVB3-3A(S11aa2) (B) and imaged by live-cell microscopy with images taken at 5-min intervals. The frames given as time zero (labeled 0) represent either an arbitrary time point (A) or the moment of appearance of the 3A-GFP signal (B). Images are presented at 180-min (A) or 10- to 20-min (B) intervals as indicated. The confocal pinhole was adjusted for analysis of local effects (95.56-µm confocal pinhole). While Golgi integrity was maintained for the duration of imaging in mock-infected cells (A), local fragmentation of GM130 was evident in infected cells (white asterisk in the GM130 channel in panel B). The onset of fragmentation was associated with a marked increase in the intensity and number of 3A-GFP punctae at the Golgi apparatus (white asterisk in 3A-GFP channel in panel B), although while 3A signal and GM130 were proximal at this stage, there was no clear colocalization (inset in panel B). Bars, 10 µm.

Enterovirus infections trigger the disassembly of the Golgi apparatus, which coincides with the formation of early ROs (18, 22). Here, we used live-cell imaging to visualize this process in living cells. To this end, BGM cells stably expressing GFP(S1-10) were transduced with murine leukemia virus (MLV) particles encoding mCherry-GM130 as a traceable marker for the Golgi apparatus. Live-cell confocal imaging was first carried out with a narrow pinhole (95.56 µm) to detect the first local changes in Golgi structure during infection. As expected, GM130 was observed as a condensed perinuclear signal in uninfected cells (Fig. 5A) or early in infection with CVB3-3A(S11aa2) when fluorescent 3A (which will be further referred to as 3A-GFP) could not yet be detected (Fig. 5B; see Movies S1 and S2 in the supplemental material). Strikingly, the first 3A-GFP signal detected in infected cells was rarely associated with GM130, but could instead be observed as distinct cytoplasmic punctae. Localization of 3A-GFP signal to the Golgi region typically occurred within 25 min (*n* = 17; range, 0 to 45 min) of initial detection. This was followed by a sharp increase in the intensity and number of Golgi-adjacent 3A-GFP punctae and a local perturbation of Golgi morphology, characterized by the onset of GM130 signal fragmentation (Fig. 5B, white asterisk in the GM130 channel). Interestingly, while this rapidly increasing 3A-GFP signal was clearly detected in the Golgi region, it rarely colocalized directly with the GM130 marker (Fig. 5B, inset), which is in agreement with previous observations in fixed cells (22). This suggests that RO membranes originate from a Golgi compartment that is not labeled by the *cis*-Golgi marker GM130 or that 3A resides there only transiently before accumulating in the ROs.

10.1128/mSphere.00104-16.4 Movie S1  Golgi disruption and 3A accumulation monitored over the course of CVB3 infection. Cropped view showing Golgi fragmentation at higher resolution (images are of the cell shown in Fig. 5B) from ~2 to 7.5 h postinfection. Fragmentation can be observed from approximately 3.5 h postinfection, and complete disassembly occurs within 30 min as evidenced by the punctate GM130 signal. The frame rate is 4 fps. Images were collected at 5-min intervals. Bar, 10 µm. Download Movie S1, AVI file, 8.3 MB. Copyright © 2016 van der Schaar et al. 2016 van der Schaar et al. This content is distributed under the terms of the Creative Commons Attribution 4.0 International license.

10.1128/mSphere.00104-16.5 Movie S2  The split-GFP system allows 3A to be monitored over the full course of CVB3 infection. Overview of a typical field of view during live-cell experiments from ~5 to 10 h postinfection (shown first with green, red, and bright-field channels and then repeated with green and red channels only). Several phases of infection can be observed, including the developing 3A-GFP signal and associated disruption of Golgi morphology, and the accumulation of ROs in the cytoplasm leading ultimately to cell lysis. The frame rate is 4 fps. Images were collected at 5-min intervals. Bar, 10 µm. Download Movie S2, AVI file, 8.1 MB. Copyright © 2016 van der Schaar et al. 2016 van der Schaar et al. This content is distributed under the terms of the Creative Commons Attribution 4.0 International license.

**FIG 6:**
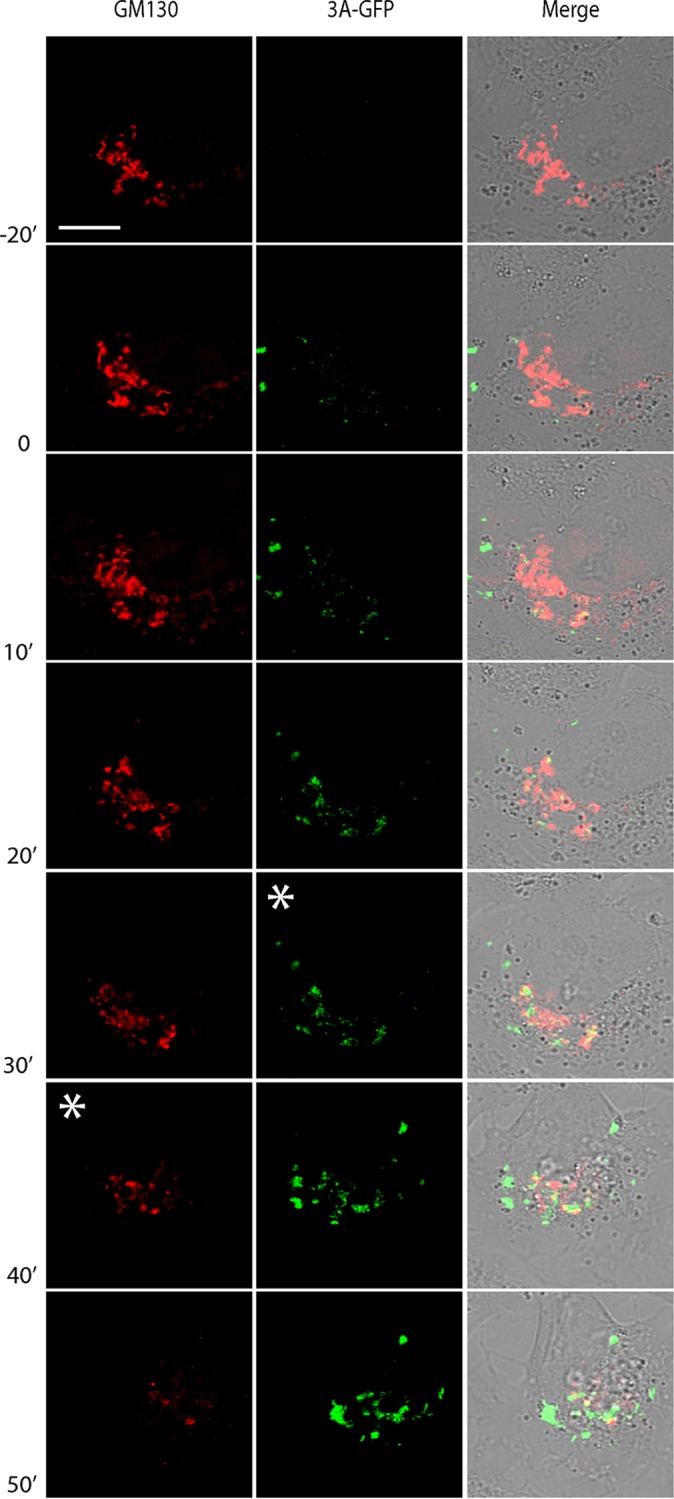
Global disruption to Golgi morphology during CVB3 infection visualized by live-cell imaging. Cells were treated as described in the legend to Fig. 5. Imaging conditions were adjusted to better ascertain global Golgi morphology (600-µm confocal pinhole) over the course of infection. Time zero (labeled 0) represents the moment of appearance of the 3A-GFP signal. Golgi fragmentation (white asterisk in the GM130 channel) was visible following accumulation of 3A-GFP (white asterisk in the 3A-GFP channel), leading to complete disassembly and a punctate GM130 signal. Bar, 10 µm.

The first signs of disruption of Golgi morphology largely began during or even preceding the accumulation of visible 3A signal (Fig. 5B, white asterisk in the 3A-GFP channel; also see Movie S1 in the supplemental material), suggesting that local changes to morphology occur rapidly and may be triggered by 3A accumulation in regions of the Golgi apparatus outside the imaging plane. To facilitate the detection of the whole Golgi complex and to monitor large-scale changes in Golgi morphology, imaging was carried out using a wider confocal pinhole (600 µm). Global Golgi fragmentation typically began 10 to 30 min after 3A started to accumulate in large amounts (Fig. 6) (*n* = 8; range, 0 to 45 min), which presumably reflects the time taken for the cumulative local changes to become apparent within the entire structure, and was completed 75 to 105 min after the initial detection of 3A-GFP signal (*n =* 8). Together, these data show that 3A accumulation coincides with the local disruption of Golgi morphology, leading to global fragmentation and eventual disassembly of the Golgi apparatus. After complete Golgi fragmentation, the 3A-GFP signal expands throughout the cell and eventually occupies the entire cytoplasm before the cell goes into demise, demonstrating that 3A-GFP signals can be imaged to the point of cell lysis (Movies S1 and S2).

### Construction of CVB3 encoding both GFP(S1-10) and 3A(S11aa2).

**FIG 7:**
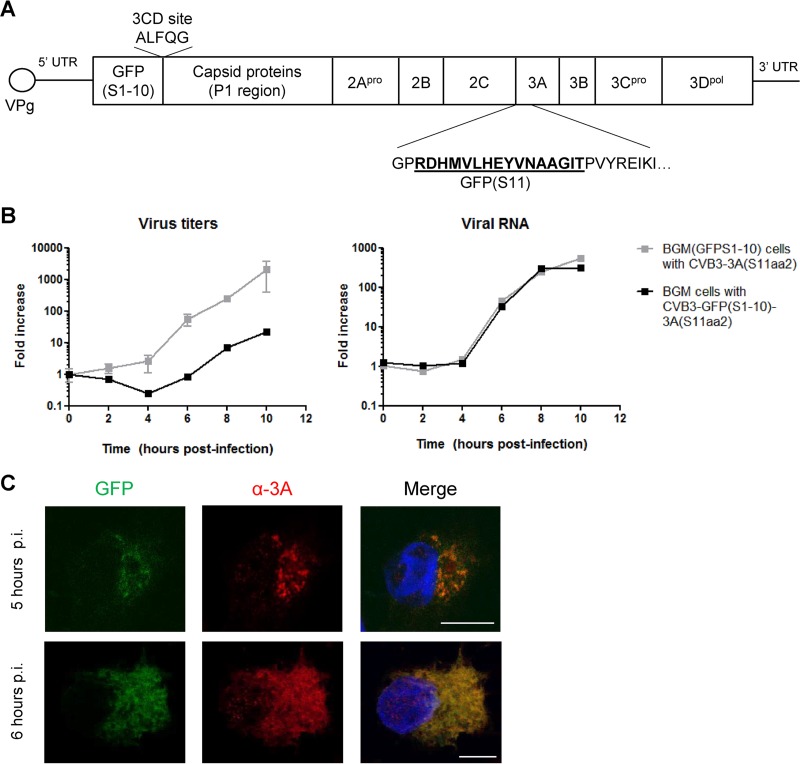
Construction of CVB3 that encodes both GFP(S1-10) and 3A(S11aa2). (A) Schematic diagram of the genome organization of CVB3 showing the insertion of GFP(S1-10) before the capsid coding region and GFP(S11) in 3A after amino acid 2. 5′ UTR, 5′ untranslated region. (B) Growth curve analysis of CVB3-GFP(S1-10)-3A(S11aa2) in BGM cells and CVB3-3A(S11aa2) in BGM(GFPS1-10) cells. Cells were infected for 30 min at an MOI of 1. At the indicated time points, cells were subjected to titration analysis after freeze-thawing cycles to determine the amount of infectious virus particles. Alternatively, cells were lysed to determine the amount of viral RNA by quantitative PCR. The results are expressed as fold induction relative to the quantities determined directly after removing the inoculum. (C) BGM cells were infected with CVB3 GFP(S1-10) 3A(S11aa2). At 5 and 6 h p.i., cells were fixed and subjected to immunofluorescence analysis. The detected GFP fluorescence resulted from the assembly of GFP(S1-10) with 3A(S11aa2). Nuclei were visualized with DAPI. Images were acquired with a Leica SPE-II DMI-4000 confocal laser scanning microscope. Typical examples of cells early in infection and later in infection are shown. Bars, 10 µm.

Studying ROs induced by CVB3 encoding split-GFP-tagged 3A in distinct cell types requires the cellular expression of GFP(S1-10). To bypass the need for delivering the GFP(S1-10) gene via retroviral transduction or by generating stable cell lines, we constructed a CVB3 that encodes not only 3A(S11aa2) but also GFP(S1-10) (Fig. 7A). To do this, the gene encoding GFP(S1-10) was inserted upstream of the capsid coding region (P1) in the infectious clone containing 3A(S11aa2). GFP(S1-10) was followed by an artificial 3CD cleavage site to release the protein from the P1 region upon translation. Viable virus [i.e., CVB3-GFP(S1-10)-3A(S11aa2)] was obtained upon transfection of BGM cells with RNA transcripts of the infectious clone. After harvesting CVB3-GFP(S1-10)-3A(S11aa2), we compared its replication kinetics in BGM cells to CVB3-3A(S11aa2) in BGM(GFPS1-10) cells, so that in both cases the 3A(S11aa2) protein would bind to GFP(S1-10). The levels of viral RNA replication were nearly identical for both viruses (Fig. 7B), suggesting that the generation of ROs during the course of infection occurs similarly. However, the virus encoding both GFP fragments [i.e., CVB3-GFP(S1-10)-3A(S11aa2)] exhibited delayed production of infectious progeny compared to CVB3-3A(S11aa2) (Fig. 7B). In line with a previous study (51), we found that the processing of the artificial 3CD cleavage site between a foreign protein and P1 is suboptimal (see Fig. S3 in the supplemental material), which may explain the delay in progeny virion production.

10.1128/mSphere.00104-16.3 Figure S3  Processing of the artificial 3CD cleavage site between a foreign protein and P1 is suboptimal. (A) BGM cells were infected with different CVB3, and lysates were prepared at the indicated time points. Cell lysates were subjected to Western blot analysis using antibodies against actin (loading control), 3A (infection control), or GFP that also recognizes GFP(S1-10). An aspecific band is indicated with an asterisk. BGM cells that stably express GFP(S1-10) showed a band at around 25 kDa, which is in accordance with the somewhat smaller size than full-length GFP (27 kDa). BGM cells infected with CVB3-GFP(S1-10)-3A(S11aa2) accumulated a clear band at around 60 kDa, which corresponds to the precursor VP0 (36 kDa) fused to GFP(S1-10). Little, if any, GFP(S1-10) was detectable, indicating a defect in processing. We also examined CVB3-EGFP (EGFP stands for enhanced GFP) (27), which also is delayed in replication compared to wild-type CVB3, but not as severely as CVB3-GFP(S1-10)-3A(S11aa2). In these samples, GFP is detected as a fusion protein to VP0 as well as an individual protein. Both results demonstrate that processing of the artificial 3CD cleavage site is suboptimal. Why cleavage of full-length GFP from VP0 appeared more efficient than for CVB3-GFP(S1-10)-3A(S11aa2) remains to be determined. Since GFP(S1-10) folds differently than full-length EGFP, this may affect processing of the cleavage site. Also, EGFP is followed by a longer linker sequence upstream of the cleavage site (ALFQG, indicated in bold type) compared to GFP(S1-10) (shown in panel B) due to cloning procedures, which may affect protein folding and processing. Download Figure S3, TIF file, 1.1 MB. Copyright © 2016 van der Schaar et al. 2016 van der Schaar et al. This content is distributed under the terms of the Creative Commons Attribution 4.0 International license.

Next, we tested whether BGM cells became GFP fluorescent upon infection with CVB3-GFP(S1-10)-3A(S11aa2). Figure 7C shows that GFP fluorescence colocalized with 3A, visualized by using an anti-3A antibody, both early and later in infection. Yet, the GFP fluorescence was substantially dimmer compared to BGM(GFPS1-10) cells infected with CVB3-3A(S11aa2). It is plausible that the dimmer signal is a result of the equimolar ratios of 3A(S11aa2) and GFP(S1-10) generated by this virus, while BGM(GFPS1-10) cells produce an excess of GFP(S1-10). Another explanation for the dim GFP fluorescence is the suboptimal cleavage of the artificial cleavage site. When GFP(S1-10) is still fused to P1, it might be unable to assemble with 3A(S11aa2) and/or become fluorescent. Nevertheless, these findings suggest that the virus containing both GFP fragments is suitable for live-cell imaging.

## DISCUSSION

Live-cell imaging is a powerful technology to gain insight into the dynamics of biological processes. In the field of virology, this method has mostly been applied to visualize the entry and egress pathways of viruses by following the fate of fluorescently labeled, individual virus particles in living cells and monitoring their interactions with cellular structures (52). To date, imaging replication structures during infection has been reported for only a few viruses, including hepatitis C virus (53, 54), vaccinia virus (55), turnip mosaic virus (56), mouse hepatitis virus (57, 58), and equine arteritis virus (59). In most of these studies, replication structures are illuminated by a viral protein that is fused to GFP. Recombinant enteroviruses that encode an RO-anchored viral protein fused to GFP (or another fluorescent reporter) have not been reported thus far. On the other hand, small epitope tags have been successfully introduced into the 3A protein of poliovirus (32), which prompted us to test whether small tags suitable for fluorescent labeling are accepted in the 3A protein of CVB3. The smallest tag for fluorescent labeling of a protein is the tetracysteine tag of 6 to 20 residues (depending on the version), but it requires visualization with biarsenical dyes FlAsH and ReAsH in extra labeling steps and results in fluorescence with a relatively poor quantum yield (60, 61). The split-GFP system uses a tag of 16 residues, GFP(S11), which has the advantage that it does not require staining but becomes directly fluorescent upon assembly with the large GFP(S1-10) fragment in living cells (34). This system has been successfully applied before in the context of a virus to study the intracellular trafficking of ribonucleoproteins of influenza A virus, where the PB2 polymerase subunit was tagged with GFP(S11) (62). In our study, we incorporated GFP(S11) into the RO-anchored 3A protein of CVB3. We show that the introduction of GFP(S11) after the second residue does not affect the localization or function of 3A when it is expressed in isolation. Whether binding of GFP(S1-10) affects the function of 3A(S11aa2) proteins remains unknown, as it is possible that not all 3A(S11aa2) proteins are bound by the GFP(S1-10) counterparts. Therefore, the subset of unbound 3A(S11aa2) proteins could be solely responsible for exerting the 3A functions.

Having found that the 3A protein was not functionally hampered by the introduction of the GFP(S11) tag, we generated a recombinant CVB3 that encodes 3A(S11aa2). Infections of this virus in BGM cells stably expressing GFP(S1-10) yielded discrete fluorescent signals. However, confirming that the fluorescent foci localize to virus-induced structures demands the higher-resolution images of subcellular structures, in the context of their surroundings, that EM can provide. Establishing this link with ultrastructure is particularly interesting in the case of enteroviruses, since their ROs can adopt various morphologies during infection (i.e., tubules, DMVs, and multilamellar structures) which, as observed by EM, often coexist. In this study, CLEM was employed to confirm that the GFP fluorescent foci localize to ROs and to establish which subset of enterovirus RO morphologies this fluorescence corresponded to. CLEM revealed that the GFP fluorescence was present at bona fide ROs, which took the form of both tubular structures and DMVs resembling those observed previously with wt CVB3 in Vero cells (18). Split-GFP CLEM thus allows the unambiguous identification of RO morphologies underlying the 3A signal and opens up new possibilities for a better understanding of the requirements for their development. This method also circumvents limitations inherent in other, similar approaches. For instance, while immunogold labeling of proteins is a popular technique that couples an electron-dense gold particle to the protein of interest, allowing it to be visualized by EM, its success depends largely upon the antibody used and the resilience of epitopes during EM sample preparation. In our experience, sample preparation procedures for immunolabeling of CVB3 3A that retain RO membranes unfortunately do so at the expense of viral epitope integrity, resulting in insufficient labeling of 3A (unpublished data).

Enterovirus ROs were imaged in living BGM(GFPS1-10) cells upon infection with CVB3-3A(S11aa2). As a proof of concept, we also expressed GM130-mCherry in these cells to visualize Golgi disassembly in real time. The onset of Golgi fragmentation was found to correspond to a period of rapid 3A accumulation in the Golgi region. Under the experimental conditions used, Golgi disruption was completed within 75 to 105 min after the first detection of 3A-GFP. Interestingly, while 3A accumulation occurred adjacent to the GM130 signal, 3A rarely colocalized with GM130-mCherry before and during Golgi disassembly. While this does not preclude the transient localization of 3A to the *cis*-Golgi, our findings suggest that ROs may be generated from another Golgi compartment not labeled by GM130, which is in line with previous observations that suggest that RO formation is initiated at the *trans*-Golgi network (22).

Enteroviruses can infect both polarized and nonpolarized cells. The use of CVB3 encoding split-GFP-tagged 3A in distinct cell types relies on the cellular expression of GFP(S1-10). As an alternative to delivering the GFP(S1-10) gene via transduction, we introduced the coding sequence of GFP(S1-10) into the viral genome together with 3A(S11aa2). This new recombinant CVB3 encoding both GFP fragments also induced GFP fluorescent foci that colocalized with 3A. While progeny virion production by this virus was delayed, the viral RNA levels were very similar to the levels in CVB3-3A(S11aa2) infection of BGM(GFPS1-10) cells, implying that the “two-fragment virus” retained its ability to form ROs. Hence, this virus would be suitable for studying enterovirus ROs in physiologically more relevant cell types, including pancreatic cells (14) and 3D-cultured CaCo-2 cells (63), without the need for ectopic expression of GFP(S1-10).

In conclusion, we have presented split-GFP-tagged CVB3 as a new tool for the direct visualization of enterovirus ROs in living cells, which will allow the study of their dynamics. Furthermore, the combination of live-cell imaging with CLEM will enable us to establish a direct link between events observed by fluorescence microscopy and morphologically distinct ROs, which could shed light onto the possible differentiated roles of these different virus-induced structures in enterovirus infection. This system will therefore serve as a valuable tool for future studies on the origin and function of enterovirus ROs.

## MATERIALS AND METHODS

### Cells.

HeLa R19, BGM (buffalo green monkey), and COS-1 cells were grown in Dulbecco’s minimal essential medium (DMEM; Lonza) supplemented with 10% fetal bovine serum and penicillin and streptomycin at 37°C and 5% CO_2_.

### Plasmids and infectious clones.

The expression construct encoding wild-type (wt) 3A, i.e., p3A-myc, was described elsewhere (64). GFP(S11), i.e., the residues RDHMVLHEYVNAAGIT, were inserted after amino acid 2 in CVB3 3A to yield p3A(S11aa2)-myc. For this, a forward primer containing the GFP(S11) tag was used in a PCR with p3A-myc as the template to generate 3A(S11aa2) as a PCR product. Wild-type 3A in p3A-myc was replaced with this PCR product using standard DNA cloning techniques. The same strategy was used to introduce GFP(S11) or the StrepII tag (i.e., residues SAWSHPQFEK) in the infectious clone of CVB3 (p53CB3/T7) described elsewhere (64), yielding p53CB3-3A(S11aa2)/T7 or p53CB3-3A(StrepIIaa2)/T7. The GFP(S1-10) expression construct pCMV-mGFP(S1-10) (CMV stands for cytomegalovirus, and mGFP stands for modified GFP) was purchased from Sandia Biotech. For the production of CVB3 encoding both GFP(S1-10) and 3A(S11aa2), the GFP(S1-10) gene followed by a 3CD cleavage site was placed directly upstream of the capsid coding region P1 in p53CB3-3A(S11aa2)/T7, similar to modifications previously described for CVB3 encoding luciferase (27). For the production of murine leukemia virus (MLV) particles, our genes of interest were cloned into the retroviral vectors pQCXIP [for GFP(S1-10) or mCherry-P4M-SidM] or pRetroQ-mCherry (for GM130), both purchased from Clontech, with standard DNA cloning techniques. In the resulting plasmids, the gene of interest is followed by an internal ribosomal entry site (IRES) and a puromycin resistance gene for the production of stable cell lines.

### Retroviral particles and the production of stable cell lines.

For the delivery of genes [i.e., GFP(S1-10), mCherry-GM130, or mCherry-SidM-P4M] into cells, we used an MLV-based retroviral vector system (Clontech). MLV stocks were produced by cotransfecting HEK293T cells with the pCAGGS-VSV-G plasmid encoding vesicular stomatitis virus protein G (VSV-G), the pMLV-Gag/Pol plasmid encoding the capsid proteins, reverse transcriptase, and integrase proteins, and the plasmid pQCXIP or pRetroQ-mCherry containing the gene of interest. Four to 5 days posttransfection (p.t.), supernatants were harvested and cleared of cell debris by centrifugation and filtration through a 0.45-µm-pore-size filter. Stocks were buffered with 10 mM HEPES, aliquoted, and frozen at −80°C for later use. Prior to transduction, the MLV particles were 5 to 10 times concentrated using a 100-kDa concentrator (Millipore). For the generation of single-cell clones stably expressing GFP(S1-10), BGM and HeLa cells were transduced and grown in the presence of puromycin (2 µg/ml for HeLa cells; 30 µg/ml for BGM cells) to generate pools of GFP(S1-10)-expressing cells, which were subsequently used to prepare single-cell clones by limiting dilution. Expression of GFP(S1-10) was monitored during passaging by immunofluorescence microscopy using the GFP antibody.

### Mammalian two-hybrid assay.

pACT, pBIND, and pG5Luc (Luc stands for luciferase) vectors were from Promega. pBIND-GBF1 encoding the N terminus of GBF1, pACT-3A, and pBIND-3A were described previously (38, 39). pACT-ACBD3 was a kind gift from J. Sasaki (Fujita Health University School of Medicine, Aichi, Japan) (45). pACT-3A(S11aa2) and pBIND-3A(S11aa2) were cloned with a forward primer containing the GFP(S11) tag using standard DNA cloning techniques. Subconfluent layers of COS-1 cells seeded in 24-well plates were transfected with 350 ng of pACT, pBIND, and pG5Luc plasmids using Fugene6 (Promega) according to the manufacturer’s protocol. At 48 h p.t., cells were lysed, and *Renilla* and firefly luciferase levels were measured using the Dual-Luciferase assay kit (Promega) following the manufacturer’s protocol. Values were converted to firefly/*Renilla* signal ratios to correct for transfection efficiencies.

### Virus infection.

The production of CVB3 from infectious clones was described previously (39). Virus titers were determined by endpoint titration according to the method of Reed and Muench and expressed as 50% cell culture infective doses (CCID_50_). BGM or HeLa cells were infected with virus for 30 min to 1 h. Following removal of the inoculum, fresh medium was added to the cells. At the time points indicated in the figures, cells were either fixed for immunolabeling, lysed to measure the amount of viral RNA by quantitative PCR, or frozen to determine the amount of infectious virus particles by titration analysis.

### Quantitative PCR.

RNA was isolated from infected cells using a NucleoSpin RNA kit (Macherey-Nagel). cDNA was synthesized using random hexamers as primers with a TaqMan reverse transcription reagent kit (Roche). The cDNA was used for quantitative PCR with the forward primer 5′-CGTGGGGCTACAATCAAGTT-3′, the reverse primer 5′-TAACAGGAGCTTTGGGCATC-3′, and the LightCycler 480 SYBR green I master kit (Roche) for 45 cycles (5 s at 95°C, 10 s at 60°C, and 20 s at 72°C) on a LightCycler 480 system (Roche).

### Western blot analysis.

Proteins were separated with 10% gradient PAGE using the Gradi-Gel gradient analysis kit (Elpis Biotech). Samples were transferred to nitrocellulose membranes (Bio-Rad). After the membrane was cut at the 15-kDa band of the marker, one membrane (proteins larger than 15 kDa) was incubated with primary antibodies against mouse monoclonal anti-β-actin (Sigma) and rabbit polyclonal anti-GFP described previously (44), which also recognizes GFP(S1-10). The membrane with proteins smaller than 15 kDa was incubated with rabbit polyclonal anti-3A described previously (44). Secondary antibodies included IRDye 680-conjugated goat anti-mouse or IRDye 800-conjugated goat anti-rabbit (LI-COR). Images of blots were acquired with Odyssey imaging system (LI-COR).

### Immunofluorescence microscopy.

BGM or HeLa cells were grown to subconfluency on coverslips in 24-well plates. Where indicated, cells were transfected with 200 ng of plasmid DNA using Fugene6 according to the manufacturer’s protocol or infected with CVB3-3A(S11aa2) at a multiplicity of infection (MOI) of 1 to 10. At 24 h p.t. or 5 to 6 h postinfection (p.i.), cells were fixed with 4% paraformaldehyde for 20 min at room temperature, followed by permeabilization with phosphate-buffered saline (PBS) containing 0.1% Triton X-100 for 10 min. Cells were then incubated sequentially with primary and secondary antibodies diluted in PBS containing 2% normal goat serum. Cellular proteins were detected with primary antibodies against PI4KIIIβ (Millipore), GBF1 (BD Biosciences), and COP-I (provided by F. Wieland, Biochemie-Zentrum, Heidelberg, Germany). GFP(S1-10) was detected with a GFP antibody, and CVB3 3A proteins were detected with a 3A antibody, both described previously (44). Alexa Fluor 488- or Alexa Fluor 594-conjugated goat anti-rabbit and goat anti-mouse (Molecular Probes) were used as secondary antibodies. Nuclei were stained using 4′,6′-diamidino-2-phenylindole (DAPI). Coverslips were mounted with FluorSave (Calbiochem). Images were acquired with an Olympus BX60 fluorescence microscope with a 40× (0.75-numerical-aperture [NA]) dry objective or a Leica SPE-II DMI-4000 confocal laser scanning microscope equipped with a 100× (1.4-NA) oil immersion objective, and the confocal pinhole was adjusted to 1 airy unit (AU).

### Live-cell imaging.

BGM(GFPS1-10) cells were seeded in glass-bottom four-chamber 35-mm dishes (CELLview) and grown to ~35% confluency before transduction with MLV mCherry-GM130 particles. Infection with CVB3-3A(S11aa2) was carried out 18 to 24 h later. Cells were washed with Fluorobrite medium (Thermo Fisher Scientific) supplemented with 8% fetal calf serum (FCS) and 25 mM HEPES just prior to imaging. Imaging was performed with a Leica SP5 confocal microscope equipped with a HyD detector and a 63× (1.4-NA) oil immersion objective, and the confocal pinhole was adjusted to 95.56 µm as the standard or 600 µm to approximate wide-field imaging. Cells were maintained in a live-cell imaging chamber at 37°C and 5% CO_2_. GFP and mCherry were excited using a 488-nm or 561-nm laser, respectively, and the appropriate emission filter, and the positions (*xyz*) were marked and imaged sequentially at 5-min intervals. For the generation of movies, live-cell imaging data were processed in ImageJ and aligned using the StackReg plugin.

### Correlative light electron microscopy.

Asymmetrical guide marks were scratched into glass-bottom eight-well chamber μ-slides (Ibidi); BGM(GFPS1-10) cells were grown to subconfluency and infected with CVB3-3A(S11aa2) on these slides. Just prior to imaging and to later aid in the correlation of fluorescent and EM images, cells were incubated with 100 nM MitoTracker deep red FM (Thermo Fisher) for 30 min, then washed several times in Fluorobrite medium supplemented with 8% fetal calf serum (FCS) and 25 mM HEPES. Imaging was carried out from ~2 h p.i. in a live-cell chamber at 37°C and 5% CO_2_ with a Leica SP8 confocal microscope equipped with a HyD detector and with a 63× (1.4-NA) oil immersion objective. For each position of interest, a low-resolution tile scan was taken of the surrounding area, including guide marks, to aid in later reidentification. Z-stacks were acquired using a 1-AU pinhole and Nyquist frequency sampling. The cells were then fixed with 4% paraformaldehyde and 2.5% glutaraldehyde in 0.1 M Sørenson’s phosphate buffer (PB) and processed for electron microscopy. The cells were postfixed first with 1% osmium tetroxide for 1 h in PB and then with 1% tannic acid in PB for 30 min. The cells were then serially dehydrated in ethanol and infiltrated and embedded in LX 112 resin (Ladd Research Industries) before polymerization at 60°C. Positions of interest imaged earlier were identified on the resin block surface through comparison with (horizontally flipped) tile scan images, the appropriate block faces were trimmed, and serial thin sections were cut and collected on copper slot grids covered with a carbon-coated formvar layer. After poststaining grids with lead citrate and uranyl acetate, image meshes spanning large areas of the grid were collected on an FEI Tecnai 20 FEG electron microscope operated at 120 kV with a charge-coupled-device (CCD) camera (US4000; Gatan) (4,000 [4K] by 4K pixel) with binning 2 and a final image pixel size of 1.94 nm. These individual images were later combined into composite stitches (65). Cells of interest were digitally extracted from their raw image files using ImageJ and Aperio Imagescope software packages for light microscope and electron microscope images, respectively. These images were then overlaid using Adobe Photoshop CS6. The mitochondrial pattern in the EM images was compared with the MitoTracker deep red FM signal to find the right transformations to correlate both types of data. While the discrepancy in the axial resolution of the light and electron microscopes limited the precision of the overlay, the use of this secondary marker was critical to determine the *z*-plane in the confocal data, the best fit of the EM cell section under analysis, and the *xy* orientation of both images, thus providing an independent marker for an unbiased localization of the 3A signal.
