# Supplementary figures and images for: Illuminating the Sites of Enterovirus Replication in Living Cells by Using a Split-GFP-Tagged Viral Protein
Source: mSphere. 2016 Jul 6;1(4):e00104-16. doi: 10.1128/mSphere.00104-16 (PMC4935779; doi:10.1128/mSphere.00104-16)

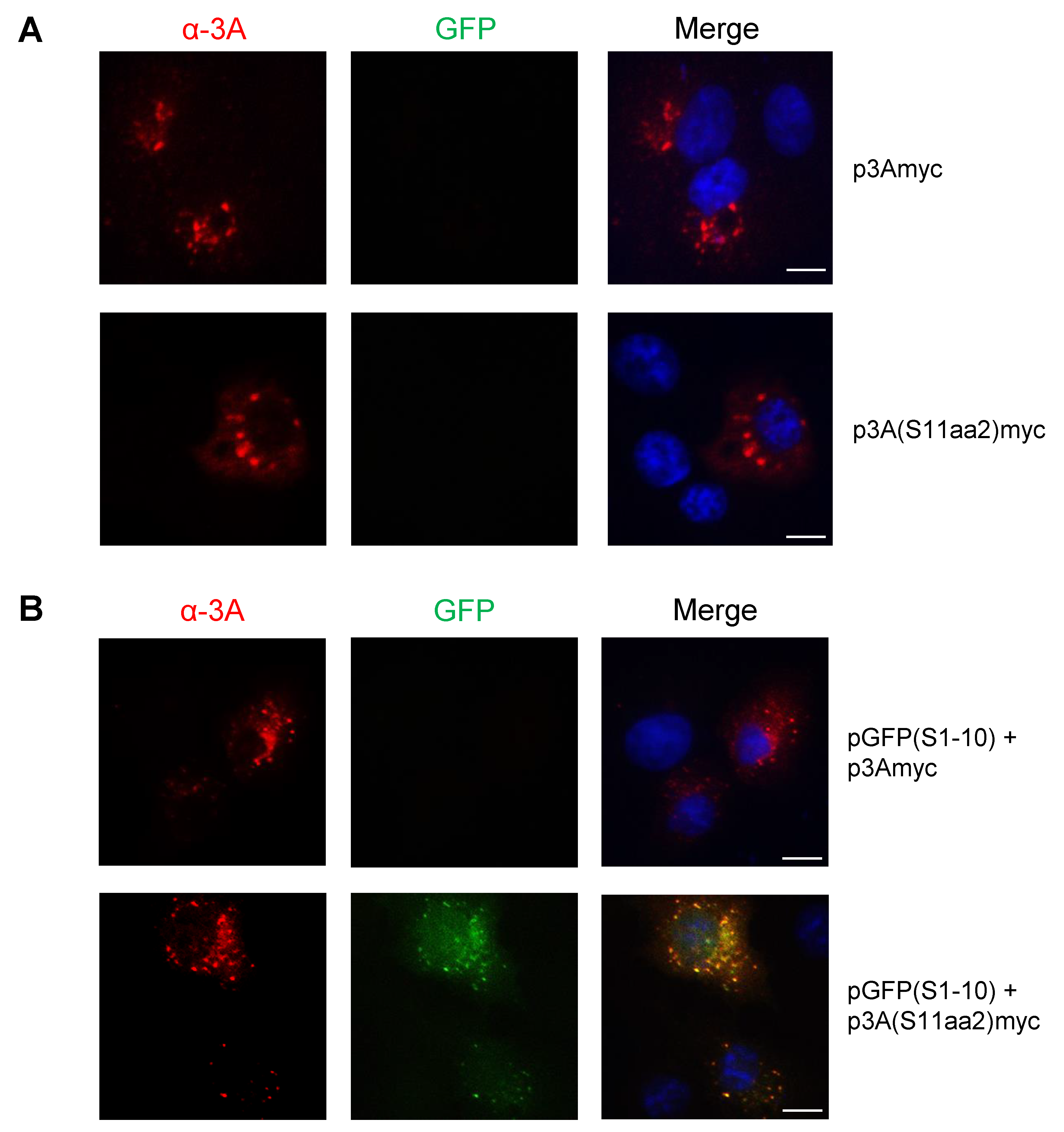

Supplement: Figure S1 [file sph004162110sf1.tif]

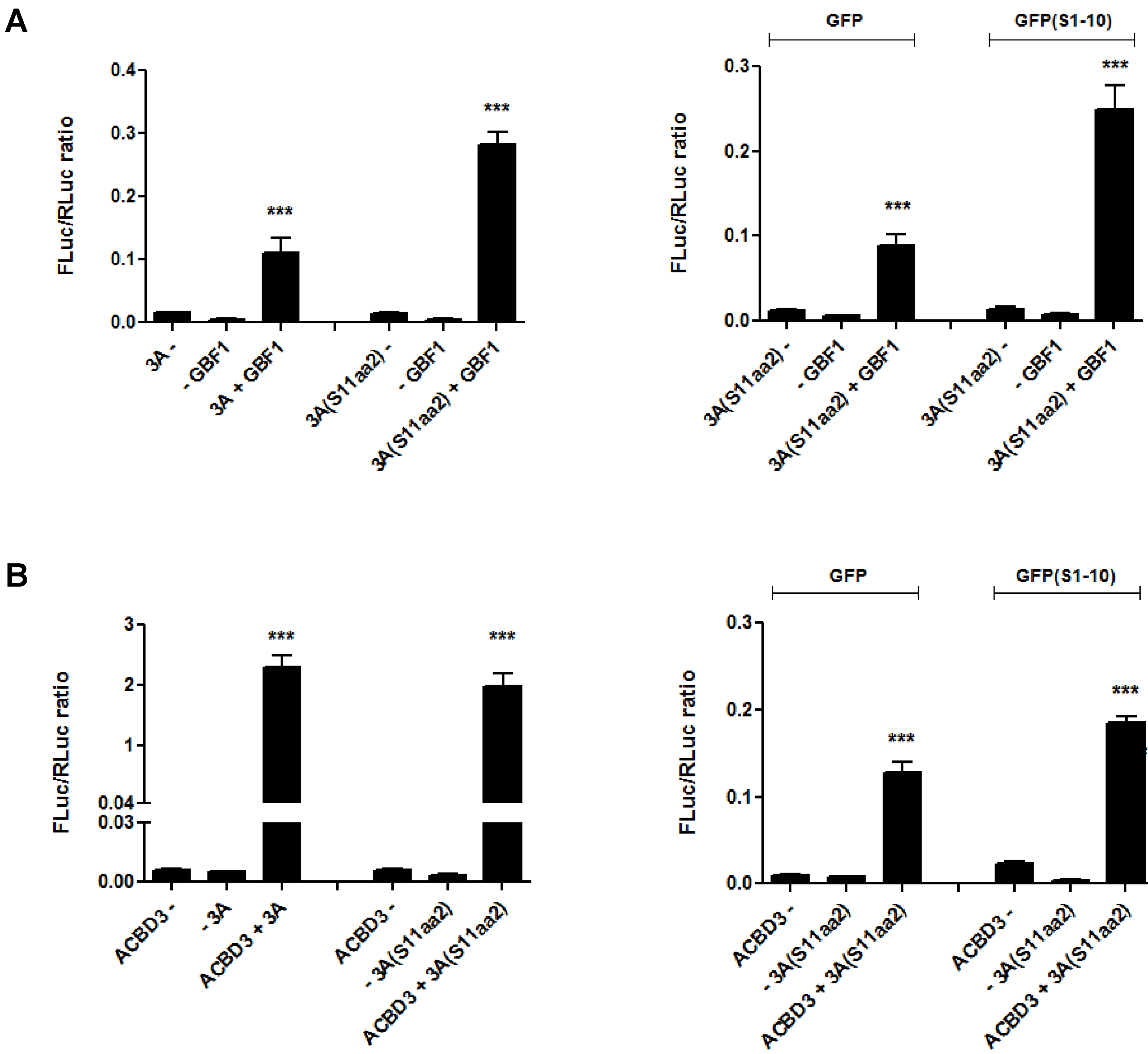

Supplement: Figure S2 [file sph004162110sf2.tif]

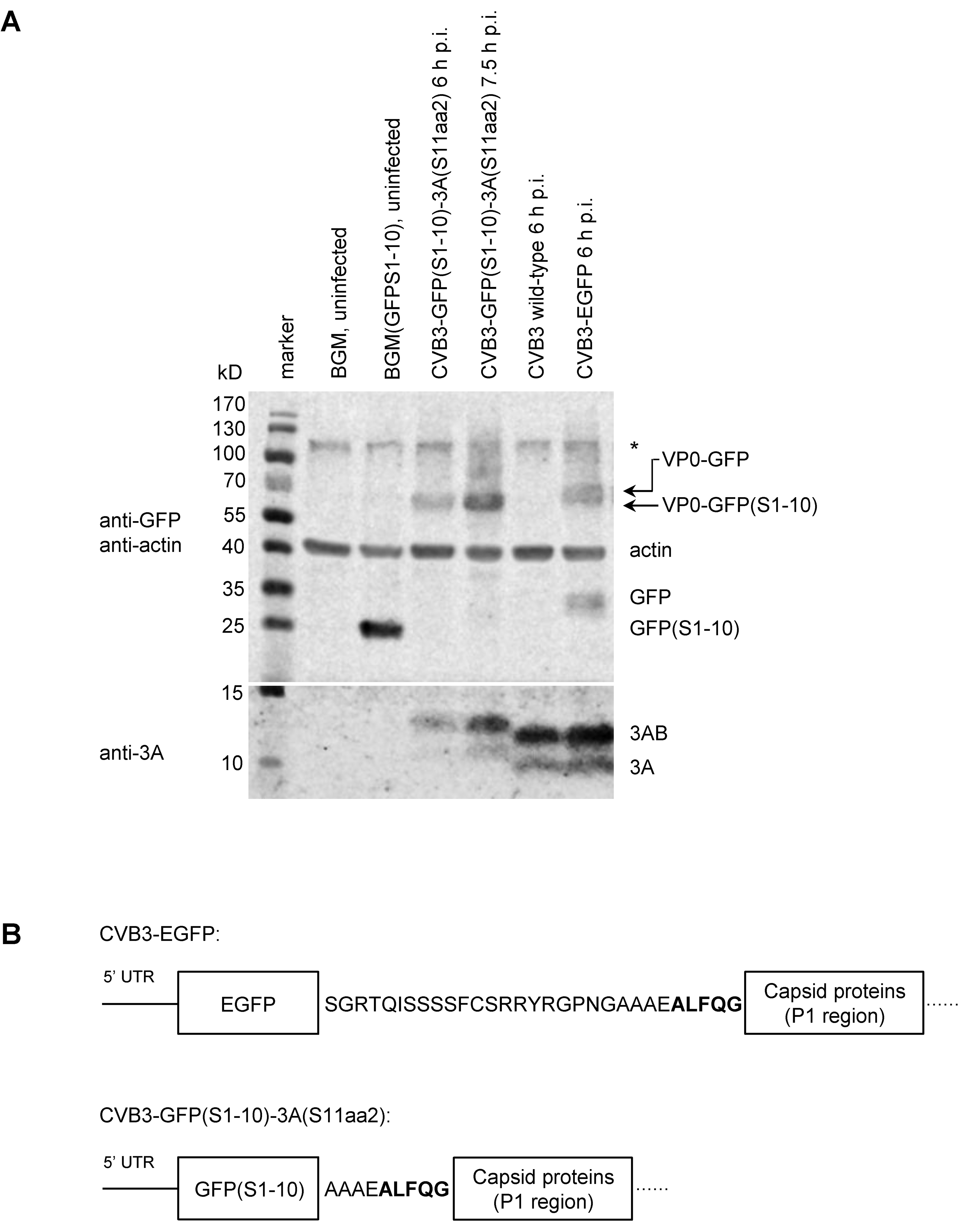

Supplement: Figure S3 [file sph004162110sf3.tif]
